# Apple Pomace in Ready-to-Eat Plant-Based Meat Analogs: Functionality, Challenges, and Opportunities

**DOI:** 10.3390/foods15122173

**Published:** 2026-06-16

**Authors:** Zibo Wang, Feifei Wang, Haizhou Wu, Jingnan Zhang

**Affiliations:** 1 Hubei Technology Innovation Center for Meat Processing, College of Food Science and Technology, Huazhong Agricultural University, Wuhan 430070, China; wzb2123836411@webmail.hzau.edu.cn (Z.W.); 18892991858@163.com (F.W.); haizhou@mail.hzau.edu.cn (H.W.); 2 Division of Glycoscience, Department of Chemistry, KTH Royal Institute of Technology, AlbaNova University Center, Roslagstullbacken 21, 114 21 Stockholm, Sweden

**Keywords:** apple pomace, plant-based meat analogs, food by-product valorization, ready-to-eat foods, refrigerated storage, whole-cut analogs, techno-economic analysis

## Abstract

Apple pomace is a widely available food processing by-product that has attracted increasing attention in circular and resource-efficient food systems for its potential in value-added food applications. The use of apple pomace in ready-to-eat (RTE) plant-based meat analogs represents a promising pathway. Unlike plant-based meats intended for cooking, RTE systems impose stricter constraints on structural stability, water retention, flavor integrity, and safety under cold chain conditions. Within this framework, apple pomace represents a compositionally complex material with both opportunities and constraints. This review examines how apple pomace and its derived ingredients can be utilized in RTE plant-based meat analogs, with particular attention to the distinct structural and functional requirements of minced-type and whole-cut products. Current evidence indicates that direct incorporation is more feasible for minced systems, where apple pomace fiber and pectin can support water retention, binding, and refrigerated slice stability when particle size, hydration, and sensory limits are controlled. By contrast, whole-cut applications are more likely to require fractionation, selective extraction, or additional structuring because particulate heterogeneity may disrupt continuous phase integrity and anisotropic structure formation. The review further identifies the main barriers to industrial translation, including water management under refrigerated conditions, flavor and color deviations, challenges in raw material standardization, and techno-economic constraints related to dewatering, processing intensity, and quality control. Overall, this review indicates that apple pomace can function as a technically relevant ingredient in RTE plant-based meat analogs. Its successful implementation depends on converting compositional complexity into predictable functionality through raw material standardization, controlled fraction use, food safety verification, and economically viable processing. In this way, sustainability-driven valorization can be better aligned with the practical requirements of industrial food production.

## 1. Introduction

By 2050, the world population is expected to approach 10 billion, while ongoing urban expansion, rising affluence, and changing food consumption patterns continue to reshape global demand for food [1]. These demographic and economic trends are intensifying concerns related to food security, environmental burden, and public health, thereby strengthening interest in plant-based foods that are nutritious, appealing, and sustainable [2]. Reflecting this trend, the growing adoption of plant-based diets has accelerated the broader plant-based foods market; Bloomberg Intelligence projected that this market could reach over USD 162 billion by 2030 [3].

RTE plant-based meat analogs constitute a category that closely resembles cold cuts, deli-style slices, and similar products, designed for direct consumption without reheating. Unlike plant-based products that require cooking prior to eating, these systems must achieve their final sensory and safety characteristics during processing, with quality maintained throughout the cold chain distribution and refrigerated storage. This distinctive requirement gives rise to four core challenges for RTE products: RTE safety, particularly microbial control and the risk of cross-contamination; cold chain and shelf life management; flavor stability, especially the amplification of off-odors and the accelerated loss of volatile compounds under refrigerated conditions; and the consistency of texture and sliceability [4,5,6].

Under current industrial practice and levels of technological maturity, minced-type products remain the most widely scaled format in plant-based meat production. U.S. retail data for 2024 indicate that patties, nuggets, tenders, wings, and ground products dominated the category, collectively accounting for nearly 80% of sales, whereas whole-cut formats represent only a small fraction, generally accounting for only about 1–5% [7]. These two formats have fundamental differences in structural requirements: minced products rely primarily on interparticle binding, water retention, and network formation, making them more tolerant to variations in raw materials, processing conditions, and batch consistency [8,9]. In contrast, whole-cut analogs must simultaneously achieve continuous phase integrity, directional anisotropy, layered architecture, and fiber bundle perception, while maintaining structural stability under refrigerated conditions without collapse, stratification, or disintegration [10,11,12,13]. Consequently, the transition from minced to whole-cut formats represents not merely a change in product form but also requires technological advances, including precise control over raw material functionality, low-temperature structuring, water migration during storage, and the stability of flavor and color, all of which are critical for successful entry into the ready-to-eat category [10,11,12,13].

When moving toward industrial production, a central question is whether a candidate plant-based meat ingredient source truly offers sustainability and scale-up potential in environmental and economic terms [14,15,16,17,18]. As a widely available and recyclable agro-industrial by-product, apple pomace offers advantages from a valorization perspective and presents a distinctive opportunity to address key formulation and processing challenges in RTE plant-based meat products [19,20]. Compared with grape pomace, citrus peel, spent grain, or tomato pomace, apple pomace is particularly relevant because it combines pectin-rich cell wall polysaccharides, insoluble fiber, polyphenols, residual sugars, organic acids, and mild fruity volatiles within the same material [19,20]. This composition makes it suitable for discussing water retention, cold structuring, oxidative stability, and sensory deviation in RTE plant-based meat systems. In the juice and food processing industries, apple pomace has become a prominent target for by-product valorization due to its compositional richness. Its high contents of cellulose, pectin, and polyphenols can contribute to structural reinforcement and functional performance in formulated systems [14]. However, the presence of residual sugars, organic acids, and volatile compounds may introduce challenges, including unintended flavor carryover and deviations from the desired sensory profile. Consequently, the incorporation of apple pomace into RTE plant-based meat products requires the resolution of several scientific questions. Accordingly, research efforts should move beyond feasibility assessments and focus on elucidating the structure–function relationships of apple pomace in plant-based meat systems. Particular attention should be given to identifying the fractions and compositional features that govern texture formation and water retention, including the roles of insoluble fiber, pectin-related network formation, and polyphenol–protein interactions across different formulations. In parallel, processing and formulation strategies must be developed to ensure structural stability, sliceability, and overall product quality under refrigerated, ready-to-eat conditions without relying on thermal setting, with emphasis on maintaining continuous-phase integrity, controlling water redistribution, and minimizing purge. In addition, apple-derived volatile and low-molecular-weight compounds require systematic investigation to enable effective management of aroma residues, mitigation of flavor deviations, and stabilization of sensory profiles, while also allowing their targeted use in formulation design [21,22].

This review examines the potential of apple pomace-derived ingredients in RTE plant-based meat analogs, with emphasis on incorporation and structuring strategies, performance limitations under refrigerated conditions, scale-up considerations from both technical and economic perspectives, and the associated food safety and regulatory requirements. By integrating these aspects, it provides a structured basis for future formulation, processing, and product design, supporting the development of applications that are sustainable, safe, and industrially feasible.

This literature relevant to this manuscript was identified mainly through Web of Science, Scopus, PubMed, ScienceDirect, and Google Scholar, using combinations of terms such as “apple pomace”, “apple fiber”, “pectin”, “polyphenols”, “plant-based meat analogues”, “ready-to-eat foods”, “high-moisture extrusion”, “cold structuring”, “life cycle assessment”, “techno-economic assessment”, and “food safety”. The search focused mainly on English-language peer-reviewed studies and relevant regulatory or institutional documents published from 2000 to 2026, with earlier sources included when they provided foundational concepts.

## 2. Composition and Functional Properties of Apple Pomace

**Figure 1:**
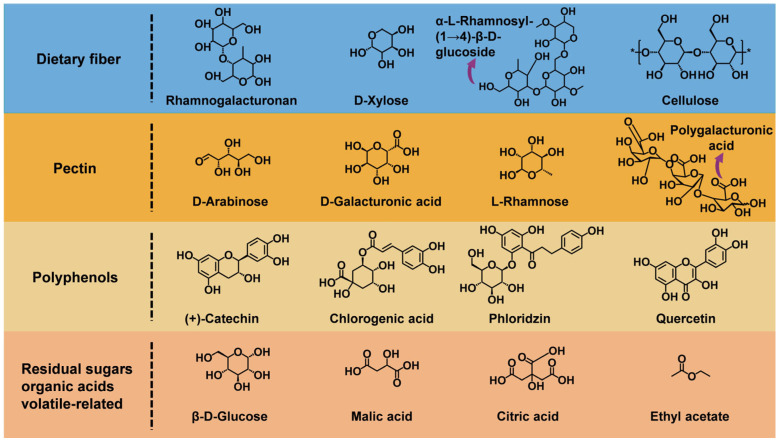
Major components of apple pomace.

Apple pomace is primarily composed of cell wall polysaccharides, residual sugars, polyphenols, organic acids, volatile compounds, and minor proximate components. However, the reported contents of these constituents are derived from different cultivars, pressing methods, drying conditions, and analytical definitions. In particular, total dietary fiber includes insoluble dietary fiber and soluble dietary fiber, while pectin is an important component of the soluble dietary fiber fraction. Therefore, the reported contents in different studies depend strongly on how each chemical category is analytically defined. Similarly, the sugar values reported in the literature may refer to reducing sugars, free sugars, soluble sugars, or soluble solids, depending on the analytical method used. Although differences in definitions and analytical methods may cause variation in the reported contents of these chemical constituents, they do not fundamentally affect the class-level mechanistic interpretation of their roles [19,20,23]. Figure 1 summarizes the major component classes and representative compounds discussed in the following subsections.

To avoid treating values obtained from different cultivars, processing histories, and analytical definitions as directly interchangeable, Table 1 collates representative reported ranges together with their sample bases and analytical methods.

### 2.1. Dietary Fiber and Pectin 

In RTE plant-based meat, the most engineering-relevant effects of apple pomace typically arise from its cell wall polysaccharide system. Mechanically, the water absorption and space filling of insoluble fiber can increase system viscoelasticity and restrict free water migration, thereby supporting chew structure and reducing exudation risk during refrigerated storage. In terms of network formation, pectin contributes to viscoelasticity. Its interactions with proteins and associated phase-separation behavior can further promote the formation of more continuous structural units, with implications for structural strength and sliceability [8].

Pectin-related effects exhibit pronounced condition dependence. Its gelation is jointly regulated by pH, ionic strength, molecular weight, degree of methoxylation, and side–chain structure; therefore, under different protein systems and cold chain conditions, even at similar addition levels, network strength and water-holding performance may differ markedly [27]. This condition dependence is particularly relevant to low-methoxyl pectin because Ca^2+^ can bridge free carboxyl groups on adjacent pectin chains, forming ionically cross-linked junction zones. In RTE plant-based meat analogs, Ca^2+^-mediated pectin networks may increase continuous phase elasticity, immobilize part of the mobile water, and support sliceable structures under low-temperature conditions, where no final heating step is available to rebuild the matrix before consumption [27,28,29]. Correspondingly, studies on plant-based meat structuring indicate that pectin may promote the formation of more muscle-fiber-like anisotropic structures by dominating or participating in phase separation, and may further affect moisture retention, fat mimetics, and flavor perception [28].

Whole apple pomace powder should not be regarded as a fully controllable structuring ingredient. It is a heterogeneous matrix containing insoluble fiber, pectin, residual soluble solids, polyphenols, organic acids, and volatile compounds; therefore, its functionality depends on how these components interact with the surrounding protein matrix. In direct incorporation, particle size, swelling behavior, polyphenol load, and protein compatibility jointly determine whether apple pomace improves water retention and sliceability or instead causes rough mouthfeel, color darkening, or flavor deviation. Thus, within whole pomace, fiber and pectin should be understood as functionally distinct contributors rather than independently adjustable modules. Whole pomace is more suitable for low-to-moderate inclusion in minced-type RTE products, whereas isolated or fractionated pectin-rich and fiber-rich ingredients are more appropriate when precise control of gelation, phase behavior, and protein–polysaccharide interactions is required [8].

### 2.2. Polyphenols

Polyphenols in apple pomace are often discussed in relation to antioxidant activity and storage stability, but their functional role in RTE plant-based meat systems is more complex. Existing studies show that flavan-3-ol, especially catechin, epicatechin, and oligomeric procyanidins, constitute important polyphenol components in apple pomace, and treatments such as hot water processing may significantly change the content of extractable polyphenols [30]. At the same time, apple cell wall materials can bind procyanidins and other phenolics, meaning that part of the phenolic fraction may remain cell wall-associated or non-extractable under routine solvent extraction [31,32]. For this reason, even if they originate from the same source of apple pomace, their antioxidant performance and reaction pathways may show obvious batch differences. Drying and processing can further change the extent of procyanidin–cell wall association, which partly explains why extractable polyphenol values vary among apple pomace samples even when the raw material source is similar [33].

A considerable part of apple polyphenols is distributed in the peel. Therefore, pomaces with relatively high peel contents have higher polyphenol concentrations compared to other parts; in fact, some by-products may still be richer in bioactive components than foods commonly eaten by humans [34,35,36,37,38]. In RTE plant-based meat systems, extractable polyphenols do not simply provide antioxidant potential. They may also interact with proteins, change protein conformation and aggregation behavior, and then influence gel-network formation, elasticity, and water-holding capacity [30,31]. Their effects can extend further to color, astringency, and volatile release, which means that the product may become more susceptible to flavor deviation or flavor drift during refrigerated storage. This issue becomes more critical in RTE products because there is no later heating step to remove some volatiles or compensate for these changes through cooked aroma development [30]. Therefore, the functional predictions related to antioxidant activity, astringency, protein interaction, and color change should be interpreted mainly in relation to the extractable polyphenol fraction, whereas cell wall-bound phenolics represent an additional source of analytical uncertainty and processing-dependent variability [33].

Accordingly, polyphenols in RTE systems should be regarded as a multidimensional quality factor rather than merely an antioxidant indicator because their contribution extends from oxidative stability to color, astringency, volatile release, and flavor stability during refrigerated storage [39,40,41,42].

### 2.3. Sugars and Organic Acids

Apple pomace, particularly the peel fraction, contains residual sugars and organic acids that can influence the sweetness–sourness balance and mouthfeel when incorporated into food products, while also serving as substrates for microbial fermentation and subsequent flavor development. This is particularly relevant for RTE plant-based meat analog systems, where fermentation can help mitigate off-flavor issues such as beany and grassy notes commonly associated with plant proteins. However, fermentation strategies developed for cookable plant-based products cannot be directly transferred to RTE systems without additional safety validation because deliberate microbial activity is introduced into a product that will not receive a later heat treatment before consumption. For RTE applications, fermentation should therefore be treated as a conditional strategy requiring validated starter cultures, defined acidification targets, control of pH and water activity, post-fermentation inhibitory conditions, hygienic handling, cold chain control, and product-specific microbiological validation [43].

In RTE systems, excessive soluble sugars and acidity may shift the product away from the expected meat-like flavor profile, while pH changes can further affect protein solubility, gelation, sliceability, and refrigerated stability. Because pectin gelation is also sensitive to pH and the solute environment, formulation should be guided by solubility and gelation characteristics, and flavor adjustment through fermentation should be balanced against microbial safety and cold chain stability; where fermentation is not used, reducing acid load through ingredient diversion or pretreatment may better limit flavor deviation and improve batch consistency [19,27].

### 2.4. Odor and Volatile Compounds 

RTE plant-based meat analogs often exhibit beany and grassy off-notes derived from legume-related volatiles, and these sensory defects are more readily perceived because the products are consumed without a heating step. In this context, apple pomace may serve as a source of aroma-active volatiles. Volatile profiling studies have shown that apple by-products, including apple pomace, contain a broad range of compounds, mainly esters and aldehydes, together with alcohols, ketones, and other compounds associated with fruity aroma characteristics [22]. These volatiles may contribute to aroma modulation, but their capacity to mask beany or grassy off-notes in RTE plant-protein matrices has not yet been validated [21,44].

The volatile contribution of apple pomace is not inherently stable because it is sensitive to raw material composition, processing history, oxidation, and microbial activity, all of which may enlarge batch-to-batch variation and promote flavor drift during refrigerated storage [35,36,37]. If apple pomace is used as an odor supplier, its application therefore requires coordinated control of raw materials, processing, and oxidation management to stabilize flavor performance during storage [21,45,46].

### 2.5. Natural Colorants

In RTE plant-based meat systems, color strongly shapes both freshness perception and consumer acceptance. Because these products are eaten without reheating, color defects are less easily concealed, and any instability of pigments or storage-related color drift tends to become more noticeable [38]. From this perspective, the color effects of apple pomace should be understood through two related but different routes. One comes from endogenous pigments retained in peel residues, mainly the background hue associated with carotenoids and anthocyanins. The other develops through browning reactions during processing and storage, where oxidation and polyphenol polymerization gradually push the hue toward yellow–brown tones [34,37]. This is why apple pomace cannot be treated too simply as a stable coloring ingredient. Its actual coloring performance depends heavily on peel proportion in the raw material, oxygen exposure, and extraction conditions, rather than on any single component alone [34,36,37].

**Figure 2:**
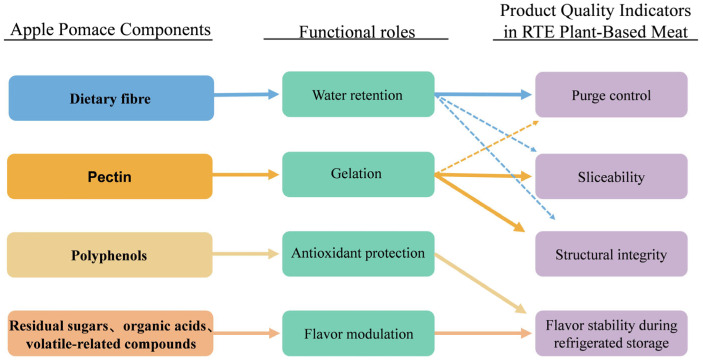
Functional roles in determining quality attributes of ready-to-eat plant-based meat analogs. Note: Solid arrows indicate primary or direct relationships, whereas dashed arrows indicate secondary or indirect relationships.

This pattern is also reflected in the available quantitative data. Total carotenoids in apple pomace are generally low, but they can still provide a certain yellow color tone. One study reported approximately 10.32 μg/g total carotenoids on a dry basis in apple pomace extracts, together with monomers such as β-carotene and lycopene [35]. The red color series appears even more limited. Anthocyanins in apple pomace powder, expressed as cyanidin-3-galactoside equivalents, were reported at approximately 4.5–7.4 mg/100 g in a specific study, which indicates that they are more likely to contribute to slight redness than to function as a primary pigment source [36]. For this reason, the current quantitative evidence still places apple pomace closer to a background color-adjustment module than to a stable primary coloring ingredient [34,37,38]. Taken together, these component classes contribute to RTE plant-based meat quality through distinct but partly overlapping functional roles, as summarized in Figure 2.

## 3. Incorporation into RTE Plant-Based Meat Analogs

The incorporation of apple pomace into RTE plant-based meat analogs primarily occurs through two pathways. One route directly introduces whole pomace or fiber-enriched fractions into formulations. In practice, this is more commonly used to adjust forming performance and water retention in minced-type products, although low-level inclusion has also been applied in whole-cut systems produced by high-moisture extrusion (HME). The other route separates apple pomace first and then adds pectin, polyphenols, and related fractions back into minced or whole-cut formulations, so that binding performance, rheological behavior, and structure formation can be regulated in a more targeted way [8,14,29,47,48,49]. These two routes also involve different techno-economic trade-offs: direct incorporation retains a lower-processing valorization pathway, whereas extraction and fractionation require additional cost but may be necessary when higher structural, sensory, and batch consistency requirements must be met [50,51].

### 3.1. Direct Incorporation

Direct incorporation introduces apple pomace into formulations as powder or slurry without prior separation. Its effects depend on component synergy and batch variability; therefore, inclusion should be guided by reproducible raw material specifications and product-specific thresholds rather than by addition level alone [8,14,22,52,53].

#### 3.1.1. Minced-Type RTE

In minced-type RTE systems, direct incorporation of apple pomace is more often used to improve cold forming and refrigerated water retention. Its main effect arises from hydration swelling and space filling of particulate insoluble fiber, which can increase viscoelasticity and restrict free water migration, thereby reducing purge during refrigerated storage and improving sliceability and forming stability [8,53]. Under some formulation conditions, commercially prepared apple fiber, when used as an enzymatically treated binder ingredient, has shown partial binding functionality together with water-holding capacity in plant-based patty systems [48]. In addition, differences in the content and state of soluble polysaccharides may alter matrix continuity and binding tendency, although such changes more often reflect amplification of batch differences at the formulation level rather than serving as a stable and reusable control lever [8,53]. Thus, the current minced-type evidence supports the potential of apple-derived fiber for binding and water retention, but it does not yet establish a universal inclusion rule for whole apple pomace, because residual sugars, polyphenols, organic acids, and volatiles may alter sensory and structural outcomes in ways not found in generic fiber studies.

Limitations that accompany these benefits mainly relate to sensory performance and batch consistency. In minced-type RTE systems, sensory compromise occurs through three main pathways. First, residual sugars and organic acids can introduce sweet–sour notes and pH shifts that move the product away from the expected meat-like profile. Second, peel-derived polyphenols may interact with proteins, increasing astringency and promoting darker color through oxidation or polymerization reactions. Third, apple-derived esters and aldehydes can create fruity flavor carryover, which is more readily perceived in cold consumption conditions and may drift during refrigerated storage. Therefore, direct incorporation should be treated as acceptable only within a formulation-specific sensory window rather than being evaluated solely by water retention or binding performance [8,14,15,22,53].

#### 3.1.2. Whole-Cut Type RTE

Whole-cut-type RTE structures rely more strongly on macroscopic continuity of the continuous phase and on a directional tearing pathway and are therefore more sensitive to the introduction of particles. When particulate features and compositional heterogeneity of apple pomace enter the system, interfacial discontinuities can form within the continuous network, shifting fracture behavior toward brittleness or a powdery mouthfeel. In one preliminary HME study, apple pomace was incorporated at 0–20%, and increasing inclusion was associated with reduced textural attributes, cutting strength, and integrity index. However, its transferability depends on the protein base, starch content, extrusion configuration, die conditions, pomace moisture, particle size, and hydration state. The observed weakening is nevertheless consistent with broader HME studies on insoluble dietary fiber, where dispersed fiber domains can interfere with protein–protein interactions, alter protein cross-linking, and change the continuity of the anisotropic matrix [14,52]. A similar pattern has also been reported in more general two-phase systems composed of proteins and insoluble dietary fiber. In such systems, fiber dispersion may weaken protein cross-linking. When the substitution level remains moderate, the system can still form a network in which the protein matrix is filled with fiber, and mechanical anisotropy may even increase. Once substitution becomes excessive, however, the structure is more likely to collapse, often together with macroscopic phase separation [52].

For whole-cut plant-based meat analogs, direct incorporation is better treated as a structural modification strategy used at relatively low inclusion levels. In this context, particle size, compatibility, and water distribution become core variables that determine whether structural continuity can be maintained. When the structural margin is already limited, additional sources of variability should be minimized as much as possible [14,22,52].

### 3.2. Extraction of Valuable Components from Apple Pomace

**Figure 3:**
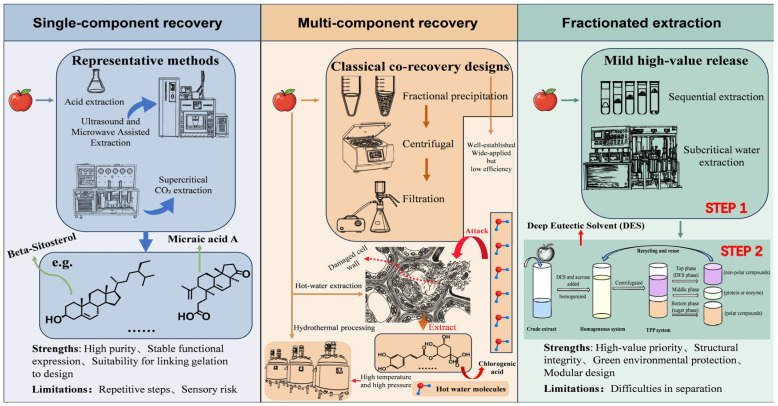
High-value recovery strategies for apple pomace. Note: The multiple dots in the figure indicate omitted representative compounds. And the colors of the water molecules are used for visual distinction only, and the colored arrows indicate direction only; neither has any additional specific meaning.

In addition to direct incorporation, extraction and fractionation provide another route for converting apple pomace into more compositionally defined ingredients. From the perspective of process routes, extraction and separation strategies can generally be divided into three types [50,54]: targeted extraction of a single component, simultaneous recovery of multiple components, and sequential extraction. The main recovery routes discussed in this subsection are outlined schematically in Figure 3.

#### 3.2.1. Targeted Extraction of Single Components

Targeted extraction becomes meaningful when a relatively clear relationship can be established between target molecules and functional outcomes because it allows apple pomace to be converted into components that are easier to formulate. Pectin is a typical example. Conventional acid extraction remains widely used and still serves as an important reference for key processing conditions [55]. At the same time, greener and more controllable routes are being developed, including strategies that combine organic acid systems with process intensification to recover pectin while linking processing parameters more closely to product physicochemical properties [56]. Non-conventional aqueous technologies have also been used to improve efficiency and reduce solvent burden. For example, ultrasound- and microwave-assisted extraction can yield usable pectin under milder conditions, while linking product metrics to end food performance [57]. This logic is not limited to pectin. Polyphenols in apple pomace are also frequently recovered as independent target fractions, but the resulting extracts should not be treated as interchangeable. Different non-conventional extraction techniques can lead to substantial differences in both yield and profile, which means that reactivity and application performance still depend strongly on the selected process [58]. Lipid-soluble small molecules can also be recovered using supercritical carbon dioxide as a solvent-friendly route. For components such as triterpenic acids and phytosterols, parameter effects, kinetics, and scale-up frameworks have been established [59]. In addition, apple pomace is increasingly being studied as a source of structural polysaccharides, including work on the analysis and recovery of xyloglucan building blocks, which supports more refined structure–function design [60].

#### 3.2.2. Simultaneous Recovery of Multiple Components

Simultaneous multi-component recovery aims to increase overall feedstock value by co-recovering pectin together with associated bioactive compounds in a single process, thereby reducing the need for repeated solvent and energy inputs. This process integration logic helps preserve the sustainability rationale of apple pomace valorization by distributing processing inputs across multiple functional fractions [61]. Its key challenge is not component release itself, but whether downstream separation and grading can support usable raw material specifications for different applications [61]. More recent hydrothermal approaches have shown that pectin, polyphenols, and sugars can be released concurrently, providing a basis for subsequent grading and application matching, although the potential gain in functional integration must be balanced against the increased sensory variability risk in RTE systems [62].

#### 3.2.3. Sequential Extraction

Sequential extraction is closer to a biorefinery logic. Typically, high-value components are released first under mild conditions, followed by sequential recovery of structural components to improve overall utilization and enhance batch consistency. Sequential processes have also been used to co-produce pectin and cellulose, allowing two structural components to be separated from the same feedstock in staged steps and giving formulation work clearer functional boundaries [63]. This idea can be extended further through solvent engineering. For example, sequential pretreatment with natural deep eutectic solvents can facilitate subsequent water-based pectin extraction while regulating key structural domains, which also shows that extraction may function not only as separation but also as modification [64]. A similar tendency can be seen in greener aqueous systems. Subcritical water extraction has been used to obtain pectin with reduced external chemical inputs, and the available results show that process conditions can regulate molecular properties as well as gel- or emulsion-related functionality [65,66]. These strategies are particularly applicable to incorporating apple pomace into RTE plant-based meat analogs because they make it easier to translate composition into reproducible functional modules and provide an industrial basis for subsequent formulation strategies involving fiber support, pectin network formation, and associated bioactive management.

### 3.3. Addition After Extraction and Fractionation

Based on the extraction and fractionation routes described above, apple pomace often shows greater practical value in RTE plant-based meat analogs when separated into more compositionally controllable extracts or enriched fractions and then added back into the system. Compared with direct use of the whole material, this route is more likely to provide stable textural performance under low-temperature processing and refrigerated storage, while also helping reduce variability in flavor and color [14,61].

#### 3.3.1. Reincorporation of Extracted Fractions in Minced-Type RTE Systems

In minced-type RTE systems, extracted fractions offer a clear advantage because they move binding and rheological control away from particle-driven water uptake and toward molecular-level network design, which substantially improves batch consistency under low-temperature processing [14,61]. This is also why pectin and soluble dietary fiber-enriched fractions often show higher formulation efficiency. Their performance depends strongly on structural parameters such as molecular weight, degree of methyl esterification, and charge characteristics, since these variables can reshape interaction pathways with salts and proteins and, in turn, influence the rate of cold gel formation and the continuity of the network. Evidence from the time-delayed cold gel concept further shows that low-methoxyl pectin can act synergistically with wheat gluten and, through regulated calcium release, support controlled gel establishment. Under low thermal load conditions, this strategy can generate a sliceable continuous structure that helps maintain forming stability in a fermented vegan sausage analog [29,67]. A similar implication can be drawn from pea protein and apple pectin composite systems, where the effects of mixing ratio on viscosity and adhesiveness suggest that this reincorporation strategy allows finer adjustment of mouthfeel and spreadability through controlled ratios, without relying on the simultaneous introduction of whole pomace components [47].

Beyond soluble fractions, insoluble fiber-enriched materials are better positioned as a controlled supplement to provide structural support and water retention. Particle size distribution and surface state provide practical entry points to reduce graininess and variability. Existing studies show that treated apple fiber can achieve acceptable binding and water-holding performance in plant-based burger systems. This indicates that fiber reincorporation is practical under controlled conditions and provides a validated basis for subsequent surface modification and formulation synergy [48]. However, this performance of apple-derived fiber or pectin-rich fractions in RTE minced systems still needs to be verified against defined protein bases, inclusion levels, particle size, storage duration, and sensory endpoints before being generalized to apple pomace as a whole ingredient.

#### 3.3.2. Reincorporation of Extracted Fractions in Whole-Cut RTE Systems

In whole-cut RTE systems, the value of post-extraction reincorporation lies mainly in reducing continuous-phase defects caused by whole-pomace particulates. Soluble fractions, especially pectin-rich fractions, are better treated as continuous-phase regulators that tune viscoelasticity, water distribution, and local phase interactions. Evidence that pectin with oil altered physicochemical properties and structural performance in high-moisture extrusion further suggests that pectic polysaccharides regulate protein phase structuring rather than directly build fibrous architecture [49]. Therefore, whole-cut formulations should prioritize extract-based forms for compatibility, while macroscopic orientation should be generated by shaping routes such as directional freezing or HME.

By contrast, insoluble fiber-enriched fractions obtained by sequential separation are more suitable for low-level reinforcement and water-locking functions, provided that particle size, dispersion, and interfacial compatibility with the oriented protein matrix are more tightly controlled because poorly integrated particles can become initiation sites for fissures and interlayer delamination [14]. This recommendation is based on the current mechanistic understanding that excessive or poorly dispersed fiber domains can disrupt protein network continuity during HME, rather than on a fully validated apple pomace-specific dosage rule. Therefore, for whole-cut RTE systems, insoluble fiber-enriched fractions should be evaluated through product-specific tests that report protein base, extrusion conditions, moisture level, particle size distribution, and storage stability. Sequential separation, therefore, helps assign pomace-derived fractions to different structural roles: soluble fractions regulate continuous-phase performance, whereas insoluble fractions should be used only for controlled reinforcement. Polyphenol and lipid-soluble extracts are more suitable at low standardized dosages to support oxidative stability without adding excessive astringency or color drift [58].

### 3.4. Cold Processing Structuring Routes

RTE plant-based meat analogs typically lack a terminal heating step that could rebuild the matrix or mask structural defects before consumption. Therefore, cold structuring routes are relevant here mainly because they determine whether apple pomace-derived fractions can be incorporated without causing purge, brittleness, flavor drift, or loss of sliceability. In this section, cold gels and composite gels, emulsion gels and structured fat systems, physical non-thermal approaches, and fermentation- or enzyme-assisted structuring are discussed from the perspective of apple pomace integration. The key question is how each route changes the functional role, optimal form, and inclusion constraints of fiber, pectin, polyphenols, residual sugars, and organic acids [68]. To clarify these route-specific differences, Table 2 summarizes the main role of each cold structuring route, the most suitable apple pomace form, and the corresponding integration constraints in RTE plant-based meat analogs.

#### 3.4.1. Cold Gels and Composite Gels

Cold gels and composite gels are relevant to apple pomace mainly when pectin-rich and soluble fiber fractions are used as continuous-phase regulators rather than when coarse whole-pomace powder is added directly. In these systems, the function of apple pomace-derived fractions is not limited to water uptake or space filling. Pectin can regulate continuous-phase viscosity, water immobilization, and network continuity, while soluble fiber may further contribute to hydration and matrix thickening. Low-methoxyl pectin is particularly relevant because Ca^2+^ can bridge free carboxyl groups on adjacent pectin chains and form ionically cross-linked junction zones. The degree of methyl esterification, molecular weight, ionic environment, and calcium release profile therefore determine whether the pectin-rich fraction behaves mainly as a viscosity modifier or as a gel-forming component [27,28,29].

For minced-type RTE systems, such cold gel or composite gel routes may improve binding, reduce purge, and support slice stability under refrigerated conditions. For whole-cut systems, however, pectin-rich apple pomace fractions should be treated mainly as continuous-phase regulators rather than as direct builders of macroscopic fibrous architecture. Directional structure still needs to be generated by shaping routes such as high-moisture extrusion, directional freezing, or other anisotropy-forming processes. The main formulation risks are heterogeneous gelation, excessive brittleness, and phase separation when pectin content, Ca^2+^ availability, pH, or ionic strength is not matched to the protein matrix. Residual polyphenols in pectin-rich fractions may also interfere with protein–polysaccharide interactions and should therefore be considered when selecting extraction and purification conditions [27,28,29,30].

#### 3.4.2. Emulsion Gels and Structured Fat Systems

Emulsion gels and structured fat systems are useful for RTE plant-based meat analogs because they can provide lubrication, juiciness, and fat-like mouthfeel without relying on a final heating step. For apple pomace, this route is more suitable for fractionated ingredients than for coarse whole-pomace powder. Pectin and soluble dietary fiber fractions can increase continuous-phase viscosity and may improve the stabilization of water and oil droplets, thereby supporting more stable emulsion gel structures. Polyphenol-rich extracts may help delay lipid oxidation, but their dosage needs to be controlled because they may also contribute to astringency, color darkening, and protein–polyphenol interactions [30,31,69,70].

In this route, insoluble apple pomace fiber should be used cautiously. Low and well-dispersed levels may improve water retention and provide structural filling, but coarse or poorly hydrated particles can create local defects in the emulsion gel network and weaken slice continuity. This limitation is especially important in RTE systems because cold consumption makes graininess, color deviation, and flavor drift more perceptible. Therefore, the optimal apple pomace form for emulsion gel and structured fat systems is pectin- or soluble-fiber-enriched fractions combined with controlled polyphenol dosing and defined particle size specifications [53,69,70].

#### 3.4.3. Physical Non-Thermal Approaches

Physical non-thermal approaches, such as high-pressure processing and high-pressure homogenization, are relevant to apple pomace-containing systems mainly because they can improve dispersion, hydration, and interfacial compatibility without introducing a significant thermal load. Their role is therefore not simply to structure plant proteins, but to reduce the defects caused by heterogeneous apple pomace fractions. For fiber-enriched apple pomace ingredients, particle size reduction and improved hydration may reduce graininess, improve water distribution, and make the fiber phase more compatible with minced-type RTE matrices. However, excessive fiber refinement may also intensify competition for water with proteins and change water migration during refrigerated storage [53,71,72].

For pectin-rich apple pomace fractions, homogenization may improve dispersion and strengthen their contribution to continuous-phase viscosity and interfacial compatibility. This can be useful when pectin is expected to support binding or stabilize dispersed oil and water domains. In whole-cut RTE systems, however, physical non-thermal treatment should be viewed as an auxiliary tool for improving compatibility rather than a primary route for forming fibrous anisotropy. Even after particle refinement, poorly integrated fiber residues may still disrupt the oriented protein network and become initiation sites for fissures or interlayer separation. Therefore, the practical value of this route depends on matching particle size, hydration state, pectin dispersion, and protein aggregation behavior to the intended product format [52,53,71].

#### 3.4.4. Fermentation and Enzymatic Cross-Linking

Fermentation and enzymatic cross-linking can support low-temperature network reinforcement, but their use in apple pomace-containing RTE systems requires careful formulation and safety control. Apple pomace contains residual sugars and organic acids that may affect acidification kinetics, sweetness–sourness balance, and microbial activity. These components may be useful when controlled fermentation is used to modulate beany or grassy off-notes, but they can also shift the product away from the expected meat-like sensory profile. Polyphenols may further modify protein gelation, increase astringency, or contribute to color darkening, especially under refrigerated storage, where no final heating step is available to mask these changes [30,31,43].

Enzymatic cross-linking provides a more controllable route for reinforcing protein networks and may help anchor reincorporated pectin or fiber fractions within the matrix. However, excessive cross-linking can produce a hard or rubbery mouthfeel, while uncontrolled addition of whole pomace may introduce variable levels of sugars, acids, fibers, and polyphenols that interfere with reproducible gel formation. For this reason, fermentation- and enzyme-assisted routes are more suitable for standardized apple pomace fractions or carefully characterized low-dose whole-pomace inclusion than for uncontrolled direct addition. In RTE applications, fermentation must also be supported by defined starter cultures, pH, and water activity targets, cold chain verification, and product-specific microbiological validation because the product will not undergo a subsequent heat treatment before consumption [9,43,73].

## 4. Technical Challenges and Solutions

### 4.1. Variability and Standardization

Apple pomace composition varies substantially across cultivars and processing chains. This variability remains evident even when attention is restricted to pectin, which is often regarded as a key determinant of structure and water retention. For example, the degree of methyl esterification can span a wide range, and such variation corresponds to differences in gelling and interfacial behaviors [25]. At the formulation level, these differences are reflected in the uncertainty of water absorption kinetics, adhesiveness, slice integrity, and purge risk during refrigerated storage. Another problem is that many existing reviews note insufficient reporting of apple pomace source information and processing history, while pretreatment conditions also differ markedly across studies. When these basic conditions are not clearly reported, cross-study comparability and engineering transferability are directly weakened. For this reason, reusable pretreatment and characterization specifications should be regarded as a prerequisite for industrialization [15].

As formulations move toward whole-cut systems, variability in both ingredients and processing becomes amplified, and HME studies show that moisture, protein concentration, and process conditions can strongly affect structure and color, requiring tighter control than in minced products. Standardization therefore must extend beyond raw materials to include in-line monitoring and process-level consistency control, for which recent near-infrared approaches provide practical routes by linking process intensity and cooling-die signals to final structuring and texture metrics [10,11,74,75,76,77].

### 4.2. Water Management and Refrigerated Structural Stability

Cold chain distribution and refrigerated shelf life place water migration at the center of structural risk in RTE products. In minced products, purge affects appearance, perceived juiciness, slice stability, and sensory consistency [8,78]. In whole-cut products, the problem is more pronounced. Whole-cut products must not only form layered structures and fiber bundle-like texture, but also maintain this anisotropic structure during refrigerated storage, avoiding interlayer separation, loosening of the matrix, and textural collapse caused by water redistribution [79,80].

Apple pomace may either support or introduce uncertainty in this respect. Fiber- and pectin-type hydrophilic polysaccharides can enhance water binding and structural support [8,28], whereas high fiber fractions may compete with proteins for water and interfere with the formation of a continuous network [14]. In HME systems incorporating apple pomace, increasing inclusion from 0 to 20% resulted in an overall decline in cutting strength and structuring-related indices, and the integrity index decreased significantly at higher inclusion levels. This structural weakening is also sensory-relevant because disrupted continuous networks and poorly integrated particles can translate into brittle fracture, powdery mouthfeel, and reduced slice integrity in whole-cut products. This has been attributed to physical disruption of the protein–starch matrix by dietary fiber and competition in water partitioning, which limits protein hydration and network development [14,52]. This creates a direct tension with the need for coordinated structural continuity and water retention during refrigerated storage in RTE products. Formulation strategies therefore require fractionation or controlled inclusion levels to balance structure and functionality.

### 4.3. Flavor and Color Changes

In RTE plant-based meats, the absence of heating makes off-flavor and color drift more noticeable during refrigerated storage. Oxidation-derived volatiles are particularly important, and studies on soy protein isolate and plant-based meat show that storage conditions can intensify off-odor formation. Refrigerated flavor stability therefore requires coordinated control of lipid oxidation, water status, and packaging rather than flavoring alone [16].

Polyphenols in apple pomace are often discussed as a basis for antioxidant and functional fortification, but the final sensory outcome still depends strongly on dosage, processing history, and matrix composition. At technologically relevant inclusion levels, the compromise in sensory quality is not a single effect. Off-flavor risk mainly arises from residual sugar–acid notes, fruity volatile carryover, and oxidation-derived volatiles during refrigerated storage. Browning or color drift is associated with polyphenol oxidation, non-enzymatic browning during processing, and oxygen exposure during cold chain distribution. Astringency is mainly linked to polyphenol–protein interactions, which can also modify gel behavior and oral perception. This does not mean that apple pomace inclusion is unsuitable, but it indicates that practical inclusion limits should be defined by sensory stability, together with water retention, oxidative stability, and texture [16,81,82]. A review of processed meat products has noted that apple and grape pomace are rich in dietary fiber and polyphenols, and that their polyphenols can inhibit lipid oxidation at levels approaching those of synthetic antioxidants. This does not mean that higher inclusion is always beneficial. Once the inclusion level rises beyond a certain point, hardness may increase, and trained assessors are more likely to detect flavor differences, including enhanced sweet and sour notes that deviate from the expected meat-like profile [15]. Empirical studies also show that the addition of apple pomace can alter color parameters and lipid oxidation levels, while microbiological indicators and shelf life safety still need to be evaluated at the same time [81]. A similar issue appears in high-moisture-extruded plant-based meat systems, where apple pomace addition also leads to overall color changes, associated both with non-enzymatic browning during extrusion and with polyphenol-related browning contributions [14].

**Figure 4:**
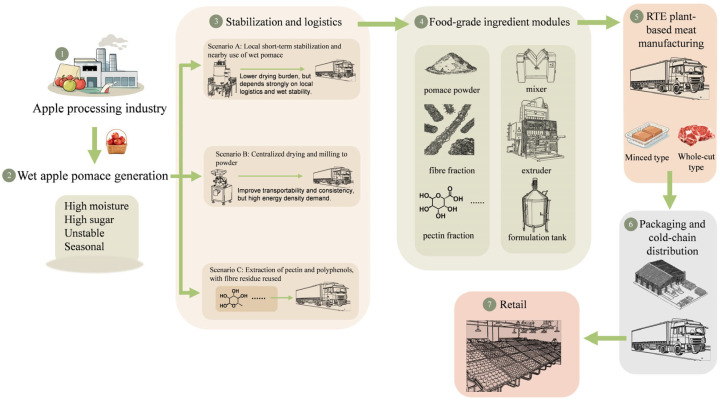
Apple pomace to ready-to-eat plant-based meat analogs value chain. Note: The multiple dots in the figure indicate omitted representative compounds.

For apple pomace-containing RTE systems, color instability should be interpreted primarily in terms of apple-derived browning pathways rather than the general colorant landscape of plant-based meats. Polyphenol oxidation can generate quinone-type intermediates that further polymerize into brown pigments, while reducing sugars and amino compounds may contribute to Maillard-type browning during drying, extrusion, or other thermal pretreatments [30,34,36]. These reactions are influenced by oxygen exposure, pH, moisture state, temperature history, and the extent of tissue disruption, so refrigerated storage can slow but not fully eliminate color drift [34,36]. Apple-derived anthocyanins and carotenoids may provide slight red or yellow background tones, but their levels in apple pomace are generally limited, and their stability is strongly affected by pH and processing conditions [35,36]. Therefore, apple pomace should be combined with more stable or complementary colorant systems and oxidation-control strategies [82]. Beyond formulation-level quality, the feasibility of using apple pomace in RTE plant-based meat analogs also depends on whether the material can be stabilized, transported, and processed with sufficient consistency and acceptable cost during scale-up. This broader scale-up pathway is summarized in Figure 4 as an apple pomace-to-RTE plant-based meat value chain.

## 5. Techno-Economic Constraints and Scaling Potential

The techno-economic feasibility of apple pomace valorization should be evaluated based on the specific properties of this feedstock rather than on generic biomass analogies. Apple pomace is seasonally generated, commonly co-located with juice processing facilities, and characterized by high initial moisture and fermentable residual sugars, which means that stabilization speed, dewatering energy, transport distance, and preservation strategy directly shape its cost profile. Recent apple pomace-specific LCA and process design studies indicate that environmental and economic performance depend strongly on the selected valorization pathway, plant capacity, energy demand, and whether high-value co-products such as pectin, phenolic fractions, or fuels are recovered within an integrated biorefinery configuration [17,50].

### 5.1. Upstream Collection, Sorting, and Supply Organization

Whether apple pomace can enter RTE plant-based meat systems depends first on whether the feedstock can be delivered to processing sites in a stable and economical way. The first barrier to scaling is therefore not simply equipment capacity, but whether a stable supply can be maintained within a controllable transport radius. Apple pomace biorefinery TEA further indicates that plant capacity and secure feedstock supply strongly affect economic viability [50].

### 5.2. Operating Costs Driven by Dewatering Energy Demand and Energy Source Structure

For apple pomace, stabilization cost is primarily a dewatering problem. Fresh pomace typically contains a high moisture content, and drying to a stable powder requires removal of most of this water before milling, storage, or food formulation. Food-grade apple pomace powder studies show that the drying method can substantially affect powder quality, while LCA studies on apple powder production further highlight the environmental importance of drying operations [83]. A reported filtration-drying energy value of 6.1–6.4 kWh per kg of water removed from high-moisture apple pomace can be used to illustrate the potential magnitude of the drying/dewatering demand, although the original study evaluated apple pomace in a solid-fuel context rather than in food-grade ingredient production [83]. Applying this value to wet apple pomace containing 75–83% moisture yields approximately 18–31 kWh per kg of dry solids equivalent. This estimate should therefore be interpreted as an indicative energy magnitude rather than a complete techno-economic estimate, as it excludes labor, equipment depreciation, grinding, packaging, storage, quality control, and compliance-related costs [83,84,85]. Apple pomace biorefinery LCA also identifies electricity and heat consumption as major contributors, confirming stabilization energy as a key scale-up variable.

### 5.3. Cost Logic of Powder Pathways and Extraction Pathways

Once apple pomace enters formulations as a powder or graded fractions, the underlying cost structure begins to resemble that of food ingredient processing, with drying, milling, grading, packaging, and storage as the main cost components. Although environmental studies cannot be read directly as cost studies, process analysis of apple powder production still makes the cost concentration easier to understand: drying tends to dominate, with low-temperature storage following behind [85]. In other words, food-oriented routes often achieve better controllability through powderization, but that gain is usually paid for through higher energy and storage inputs. Under these conditions, scaling of powder pathways depends less on building ever more complex grading systems and more on whether equipment utilization, specific dewatering energy consumption, and cold chain efficiency can be kept in coordination.

Extraction pathways follow a process-economic logic in which profitability is shaped by yields, separation intensity, and utility burdens. For apple pomace pectin recovery, the economic pressure comes from extraction, clarification, concentration, precipitation, solvent handling, and drying of the final fraction. A small-scale input-based estimate reported a production cost of approximately Rs. 808.30 per kg of pectin from apple pomace, with precipitation medium cost identified as a major contributor; this value should not be treated as an industrial benchmark, but it indicates the sensitivity of pectin recovery to solvent use and downstream separation [86]. More recent forward-osmosis-assisted concentration work showed that replacing or reducing thermal concentration before pectin recovery can lower both capital investment and operating cost, shortening the payback period from more than five years to about two years at 80% water recovery [51]. These findings support the conclusion that pectin fraction recovery becomes more feasible when dewatering and concentration are integrated into the process rather than added as isolated unit operations. Economic feasibility becomes less straightforward when higher yields require costly unit operations. Apple pomace biorefinery studies similarly show that enzyme loading and conversion efficiency directly affect total cost [87]. Thus, extraction pathways are scalable only when the added yield justifies separation, purification, and utility costs while maintaining repeatable operation after scale-up [51,87,88]. For polyphenol-oriented valorization, subcritical water extraction of apple pomace has been reported as more profitable than ethanol extraction or anaerobic digestion alone [54].

### 5.4. Quality Testing, Cold Chain, and Compliance as Preconditions for Scaling

Compliance-related investment should be treated as a fixed operational requirement rather than as a marginal add-on after formulation development. When apple pomace-derived powders, extracts, or enriched fractions are used in chilled RTE products, cost is added through raw material screening, batch traceability, microbiological and contaminant testing, validated stabilization, hygienic storage, documentation, and cold chain verification. These requirements do not determine product functionality directly, but they determine whether a low-cost by-product can be converted into a food-grade ingredient with reproducible quality at scale. The detailed food safety and regulatory basis for these controls is discussed in Section 6 [5,89].

### 5.5. Three Most Critical Parameters in Cost Sensitivity

Based on the apple pomace-specific evidence discussed above, the most cost-sensitive parameters can be grouped into three categories. The first is stabilization intensity, especially the energy required to remove water from wet apple pomace and convert it into a food-grade powder or intermediate ingredient. Because fresh apple pomace is high in moisture and biologically unstable, delayed stabilization can increase both quality loss and processing cost [83,84,85]. The second is the recovery yield relative to the downstream separation burden. Pectin-, polyphenol-, and fiber-rich fractions become economically meaningful only when the value of the recovered ingredients can offset extraction, clarification, concentration, solvent handling, drying, and quality control costs [51,61]. The third is the integration of the supply chain with juice processing facilities. Co-location or near-source stabilization can reduce the handling of unstable wet pomace and may allow better use of existing heat, water, labor, and analytical infrastructure [17,50]. Therefore, the scale-up potential of apple pomace in RTE plant-based meat analogs should not be inferred from by-product availability alone, but from whether dewatering, fraction recovery, food-grade control, and product functionality can be integrated into a single economically coherent processing chain.

## 6. Food Safety and Regulatory Compliance

Food safety control for apple pomace-derived ingredients should begin before final RTE product manufacture. Fresh wet apple pomace is a high-moisture, sugar-rich, and biologically unstable matrix, making it susceptible to microbial growth, fermentation, spoilage, and odor-forming biochemical changes during delayed stabilization, storage, and transport. Therefore, raw material selection, exclusion of damaged or moldy apple lots, rapid stabilization, hygienic handling, time–temperature control, moisture or water-activity reduction, and batch traceability should be treated as upstream control points [19,56,90].

### 6.1. Risk Management of RTE Products

RTE plant-based meats lack a terminal heat step for microbial inactivation. Risk management therefore must be shifted upstream to process and environmental control and relies on cold chain integrity to maintain shelf life safety. For refrigeration-associated hazards such as Listeria, the U.S. Food and Drug Administration (FDA) and the U.S. Department of Agriculture, Food Safety and Inspection Service (USDA FSIS) guidance for RTE foods emphasizes environmental hygiene, segregation to prevent cross-contamination, and continuous monitoring and verification. By distinguishing hygienic zones from non-hygienic zones and strengthening sanitation and environmental monitoring, risks are maintained under continuous control throughout the process [4,6]. Cold chain failure scenarios should be treated with explicit caution, because refrigeration does not mean zero microbial growth, and the combination of temperature elevation and extended storage time can further amplify risk levels [6]. Codex and ISO 22000 provide the broader hygiene, HACCP, verification, and continual-improvement framework for this control logic [5,91].

### 6.2. From Food By-Product to Food Ingredient

When apple pomace enters the food chain as a by-product, compliance issues mainly center on food-grade requirements for the raw material, potential contaminants, and the permissible boundaries of labeling and claims. Pesticide residues and contaminants need to remain within regulatory limits, with batch traceability and risk-based, tiered sampling in place to support control. In this respect, the European framework for maximum residue levels and the regulation on maximum levels of contaminants provide clear regulatory boundaries [92,93]. Labeling and claims should likewise be based on verifiable composition testing and restricted to what the regulations permit. Regulatory assessment should also be extended beyond the EU context. In the United States, apple pomace-derived powders, extracts, or enriched fractions should be evaluated according to their identity, intended use, and manufacturing process. If a fraction is intentionally added to food and affects the characteristics of the final product, its regulatory pathway may involve the food additive framework under 21 CFR Part 170, unless the specific use is generally recognized as safe under the FDA GRAS framework [94,95]. In China, food-grade apple pomace ingredients would need to comply with national food safety requirements, including contaminant control under GB 2762 and labeling of prepackaged foods under GB 7718. If the fraction has no traditional consumption history in China, is isolated from plant material in a new form, or has undergone processing that substantially changes its structure or composition, assessment as a new food raw material may also be required before food use [94,96]. European regulations on nutrition and health claims and on consumer food information define the limits of permissible expressions, while Codex guidance further stresses that claims should be supported by scientific evidence and should not be misleading [97,98,99]. Residual sugars in apple pomace may also affect nutrition labeling and product positioning in savory RTE products. Under 21 CFR 101.9, total sugars and added sugars must be declared in the United States, while EU Regulation 1169/2011 requires carbohydrates and sugars to be included in the mandatory nutrition declaration [95,98]. Therefore, apple pomace should not be treated only as a fiber-rich ingredient because its residual glucose, fructose, and sucrose may increase labeled sugar values and alter consumer perception in deli-style products. Desugaring by washing, enzymatic hydrolysis, or fermentation may reduce sugar contribution, but it can also change flavor, browning behavior, microbial stability, and structuring performance [43,84]. Two apple pomace-specific chemical hazards also require explicit control before food-grade use. The first is patulin, a mycotoxin associated with mold-damaged apples and regulated in apple-derived products. Under EU Regulation 2023/915, maximum levels are set at 50 μg/kg for fruit juices and apple-derived fermented drinks, 25 μg/kg for solid apple products placed on the market for final consumers, and 10 μg/kg for apple products intended for infants and young children; the U.S. FDA also uses 50 μg/kg as the regulatory action level for patulin in apple juice, apple juice concentrates, and apple juice products [100,101]. Although these limits are not written specifically for apple pomace powder used in RTE plant-based meat analogs, they provide a practical reference for procurement specifications and batch screening when apple-derived streams are converted into food ingredients. Operationally, moldy or damaged apple lots should be excluded, wet pomace should be stabilized rapidly, and patulin testing should be included when raw material history or storage conditions are uncertain [100,102]. The second hazard is seed-derived cyanogenic glycosides, mainly amygdalin. Apple seeds have been reported to contain approximately 1–4 mg/g amygdalin, and cyanogenic glycosides can release hydrogen cyanide after tissue disruption or enzymatic hydrolysis; therefore, seed retention in pomace streams should be controlled during pressing, drying, and milling [103,104]. For food-grade apple pomace fractions, seed content should be specified, seed-rich streams should be separated when possible, and total cyanogenic potential should be assessed when retained seed material is likely to enter concentrated or finely milled fractions [103,104].

For RTE products, a combined barrier strategy is often the more feasible engineering route. When formulation, processing, packaging, cold chain, and monitoring are integrated as multiple barriers, system-level risk caused by failure at a single control point can be reduced [105]. Under this framework, monitoring and verification capabilities become critical because they determine whether control can be demonstrated rather than merely assumed. ISO IEC 17025 sets out general requirements for laboratory competence and result reliability, and can therefore be used as a basis for assessing the credibility of the monitoring system [89]. Allergen risk should also be considered when apple pomace or apple pomace-derived fractions are used in RTE products. Apples contain several allergenic proteins, including the Bet v 1 homolog Mal d 1 and the non-specific lipid transfer protein Mal d 3. Mal d 1 is mainly associated with birch-pollen-related apple allergy and is generally heat- and proteolysis-sensitive, whereas Mal d 3 is mainly peel-associated and is more resistant to heat and pepsin digestion; therefore, thermal treatment or drying cannot be assumed to eliminate all apple-related allergenic risk, especially in peel-rich pomace fractions [106,107,108]. Extraction and fractionation may reduce allergen loads if proteinaceous residues are removed during purification, but this should be verified analytically rather than assumed from the process route alone. From a labeling perspective, apple is not currently listed as a mandatory major allergen under EU Regulation 1169/2011 Annex II or under the U.S. FALCPA/FASTER major allergen framework, although apple pomace should still be declared as an ingredient when used. In China, allergen and ingredient labeling should be checked against the applicable GB 7718 requirements, and voluntary risk communication may be needed when apple-derived fractions are used in products marketed to sensitive consumers [98,109].

### 6.3. Novel Food Implications of Fractionated Apple Pomace Ingredients

Fractionated apple pomace ingredients also raise a separate regulatory issue because their status depends on both the source material and the processing route. Whole apple pomace powder produced by conventional drying and milling may be easier to justify as a food by-product ingredient when food-grade quality, contaminant limits, and labeling requirements are satisfied. By contrast, fractions obtained through subcritical water extraction, deep eutectic solvent pretreatment, or isolation of less conventional polysaccharides may require additional assessment because these processes can alter composition, molecular structure, residual processing aids, impurity profiles, and expected intake. Under EU Regulation 2015/2283, a food may be considered novel if it was not consumed to a significant degree in the EU before 15 May 1997 or if it results from a production process not previously used for food production in the EU that causes significant changes in composition or structure [110]. EFSA also recommends requesting pre-submission advice at an early stage to clarify the requirements and content of a future novel food application [111]. Therefore, subcritical water pectin extracts, deep eutectic solvent-derived fractions, and isolated xyloglucan-rich fractions should not be assumed to share the same regulatory status as conventionally dried apple pomace. Their market entry should be evaluated on a case-by-case basis based on source, process, composition, exposure, and toxicological evidence.

## 7. Future Perspectives

Future research should move from feasibility claims toward testable structure–function rules for apple pomace-derived ingredients in RTE plant-based meat analogs. A key question is whether insoluble fiber, pectin-rich fractions, and polyphenol-rich fractions can be assigned to reproducible formulation roles across protein matrices. This requires parallel reporting of particle size, pectin degree of methyl esterification, molecular weight, polyphenol load, protein base, inclusion level, and refrigerated storage conditions [27,28,30].

Whole-cut applications require stricter validation than minced-type systems. Current evidence suggests that whole pomace is more suitable for low-level modification in minced products, whereas whole-cut analogs may require fractionated ingredients to maintain continuous-phase integrity and anisotropic tearing. Future studies should test this assumption under defined HME conditions, including protein base, moisture content, screw configuration, cooling-die conditions, particle size, and storage time [10,14,52,79].

Future studies should also treat flavor, color, safety, shelf life, and consumer acceptance as linked outcomes. Residual sugars, organic acids, polyphenols, and volatiles may jointly affect sweetness–sourness balance, browning, astringency, aroma drift, microbial stability, and sensory rejection during refrigerated storage. Therefore, future work should combine instrumental analysis, trained sensory panels, consumer hedonic testing, willingness-to-pay analysis, and label language experiments to determine whether apple pomace-containing RTE products can meet both technical and market expectations [16,34,36,79,112,113,114,115,116].

## 8. Conclusions

Apple pomace is a promising ingredient for ready-to-eat (RTE) plant-based meat analogs, but its value lies not only in by-product utilization. As discussed in this review, its relevance to RTE systems arises from the combined roles of fiber, pectin, polyphenolic components, residual sugars, organic acids, and volatile-related fractions in shaping structure, water retention, flavor, color, and refrigerated stability, while these effects remain strongly dependent on compositional variability, incorporation route, and processing conditions. The available evidence suggests that direct incorporation is more suitable for minced-type RTE products when raw material specifications and inclusion levels are well controlled, whereas fractionated or extracted components appear more advantageous for whole-cut systems because of their higher demands on continuous-phase integrity and structural stability. At the same time, stable and transferable relationships between fraction characteristics and final product performance remain insufficiently established, especially with regard to structural stability, sliceability, flavor quality, color stability, and oxidative control under refrigerated conditions. Overall, this review clarifies the key conditions under which apple pomace can move from a promising agro-industrial by-product to a technically meaningful ingredient in RTE plant-based meat analogs, thereby providing a stronger basis for future product development and process design toward sustainable, safe, and practically scalable applications.

## Figures and Tables

**Table 1:** Reported compositional ranges, sample sources, and analytical bases of major apple pomace components.

Component Category	Representative Reported Range	Source	Analytical Method or Standard Reported
Total dietary fiber	33.40–51.85 g/100 g dry apple pomace; other studies report approximately 35–52.9% dry weight	[24,25]	Commonly reported using enzymatic-gravimetric dietary fiber methods, such as AOAC 985.29 or related AOAC/AACC dietary fiber methods when specified
Insoluble dietary fiber	Source-specific values should only be interpreted within the same study that reports total, soluble, and insoluble fractions	[24]	AOAC/AACC enzymatic–gravimetric methods, when specified
Soluble dietary fiber and pectic polysaccharides	Pectin content reported as 7.36% in one apple pomace flour study; pectin-related galacturonic acid reported at 11.8–21.6% in mono-varietal cider apple pomaces	[26]	Pectin extraction and characterization methods vary; galacturonic acid, degree of methyl esterification, and molecular properties are often measured separately
Free or reducing sugars	Sato et al. reported residual sugars as a major fraction, with an average value around 54.4% in dried pomace from eleven cultivars	[24]	Method-dependent; reported as reducing sugars, free sugars, soluble sugars, or soluble solids depending on source
Protein	Approximately 1.2–4.7% dry basis in review sources; Sato et al. reported cultivar-dependent protein values around 3.75–4.65%	[19,20,24]	Nitrogen-based methods, such as AACC 46-30.01 or combustion/Kjeldahl-type methods, when specified; O’Shea-related methods used a fruit/vegetable nitrogen-to-protein factor of 5.70
Lipids	Approximately 0.6–4.2% dry basis in review sources	[19,20,24]	Usually, solvent extraction/Soxhlet-type methods when specified by the original source
Ash or minerals	Source-specific; not used here as a universal additive range	[19,20,26]	AOAC 923.03 for ash when specified
Organic acids	Source-specific; often reported as titratable acidity or malic acid equivalent	[24]	Titratable acidity, HPLC, or equivalent acid analysis, depending on source
Extractable polyphenols	Sato et al. reported total phenolics at 2.29–7.15 g/kg dry pomace, expressed as catechin equivalents	[24]	Folin–Ciocalteu assay or solvent extraction-based phenolic analysis

Note: For formulation use, the key variability drivers are component-specific. Pectin content and pectin degree of methyl esterification are primarily affected by cultivar group and press type, with additional effects from peel-to-pulp ratio, drying history, and extraction conditions. Extractable polyphenol content is mainly influenced by cultivar group, peel enrichment, tissue disruption, drying or heat exposure, and extraction protocol. Residual soluble sugars are mainly governed by cultivar maturity, juice extraction efficiency, press type, and washing, fermentation, or other desugaring pretreatments.

**Table 2:** Cold structuring routes and apple pomace-specific integration constraints in RTE plant-based meat analogs.

Cold Structuring Route	Main Role in RTE Structuring	Suitable Apple Pomace Form	Main Integration Constraints
Cold gels and composite gels	Low-temperature network formation and water immobilization	Pectin-rich and soluble-fiber fractions	Pectin degree of methyl esterification, molecular weight, Ca^2+^ availability, pH, ionic strength
Emulsion gels and structured fat systems	Juiciness, lubrication, lipid structuring, and water/oil stabilization	Pectin, soluble fiber, low-dose polyphenol-rich fractions	Interfacial compatibility, polyphenol-induced darkening, astringency, fiber particle defects
Physical non-thermal approaches	Dispersion improvement, particle refinement, and interfacial compatibility	Fiber-enriched fractions and pectin-rich fractions	Particle size, hydration state, excessive water competition, protein aggregation
Fermentation and enzymatic cross-linking	Acid-induced structuring, flavor modulation, and network reinforcement	Standardized fiber/pectin fractions; controlled low-dose whole pomace only after validation	Residual sugars, organic acids, polyphenol–protein interactions, RTE microbial safety validation

## Data Availability

No datasets were generated or analyzed during the current study.

## Abbreviations

The following abbreviations are used in this manuscript:

RTE	Ready-to-eat
PBMA	Plant-based meat analog
HME	High-moisture extrusion
TEA	Techno-Economic Assessment
FDA	U.S. Food and Drug Administration
USDA FSIS	U.S. Department of Agriculture Food Safety and Inspection Service
HACCP	Hazard Analysis and Critical Control Points
ISO	International Organization for Standardization
ISO/IEC	International Standard Specifying General Requirements for the Competence of Testing and Calibration Laboratories
EU	European Union
